# Smoking-Related Health Beliefs in a Sample of Psychiatric Patients: Factors Associated with the Health Beliefs and Validation of the Health Belief Questionnaire

**DOI:** 10.3390/ijerph18041571

**Published:** 2021-02-07

**Authors:** P. V. Asharani, Jue Hua Lau, Vanessa Ai Ling Seet, Fiona Devi, Peizhi Wang, Kumarasan Roystonn, Ying Ying Lee, Laxman Cetty, Wen Lin Teh, Swapna Verma, Yee Ming Mok, Siow Ann Chong, Mythily Subramaniam

**Affiliations:** 1 Research Division, Institute of Mental Health, Singapore 539747, Singapore; Jue_Hua_LAU@imh.com.sg (J.H.L.); Ai_Ling_SEET@imh.com.sg (V.A.L.S.); Fiona_Devi_SIVA_KUMAR@imh.com.sg (F.D.); peizhi_wang@imh.com.sg (P.W.); K_ROYSTONN@imh.com.sg (K.R.); Ying_Ying_LEE@imh.com.sg (Y.Y.L.); Laxman_CETTY@imh.com.sg (L.C.); Wen_Lin_TEH@imh.com.sg (W.L.T.); Siow_ann_chong@imh.com.sg (S.A.C.); mythily@imh.com.sg (M.S.); 2 Early Psychosis Intervention Programme, Institute of Mental Health, Singapore 539747, Singapore; Swapna_verma@imh.com.sg; 3 Office of Education, Duke-NUS Medical School, 8 College Road, Singapore 169857, Singapore; 4 Department of Mood and Anxiety, Institute of Mental Health, Singapore 539747, Singapore; Yee_ming_mok@imh.com.sg

**Keywords:** risk perception, health beliefs, health cognition, smoking, mental illness

## Abstract

This study examined the (a) health beliefs and emotions (perception of risk, benefits, severity, and worry) about smoking among current and former smokers, (b) their awareness of health warnings, (c) factors associated with smoking-related health beliefs, and (d) the factor structure of the health belief questionnaire. Participants (n = 184) were recruited from a tertiary psychiatric care hospital. Current smokers showed a significantly higher risk perception and lower perceived benefits compared to former smokers. Younger age (<40 years), nicotine dependence (ND), a history of smoking-related diseases (SRD), and intention to quit were significantly associated with a higher risk perception in current smokers. Younger age, a history of SRDs, and motivation to quit were positively associated with health beliefs, while the latter two were associated with worry. Motivation and younger age were associated with a better perception of benefits and severity. Information on the cigarette packets was the major source of awareness for the sample, and 69% reported that existing campaigns were not effective in discouraging their smoking. Personalized risk communication and educational initiatives must focus on improving the knowledge of risk, benefits, and increase motivation to promote health cognition and thus smoking cessation.

## 1. Introduction

Smoking is an enormous public health burden, with 1.1 billion adults smoking tobacco products globally and more than 8 million people dying due to smoking-related diseases (SRDs) every year [1,2]. SRDs such as cancer, cardiovascular diseases, pulmonary diseases, endocrine disorders, and infertility are major contributors to mortality and morbidity among smokers [3]. On average, smokers die at least 10 years earlier than non-smokers, and smoking cessation reduces this risk by 90% [4]. Many countries have adopted stringent control measures to reduce the supply and demand for tobacco products and promote smoking cessation. Despite these aggressive tobacco control measures, the prevalence of tobacco use and SRDs have remained high in the past decade [5].

The global prevalence of tobacco use was found to be 24.9% [6]. Southeast Asia (SEA) has a higher prevalence of smoking, with 31.2% of the population being smokers. SEA has reported the highest prevalence of smoking (50% of the global burden) compared to other regions. A study from SEA showed that a lower perception of smoking-related consequences was a significant determinant of smoking initiation in adolescents [7]. The perception of smoking-related consequences plays a vital role in an individual’s decision to continue smoking [8]. The Health Belief Model (HBM) suggests that a person’s health-related behaviour changes based on six factors: (a) susceptibility: the perceived vulnerability towards the disease/condition; (b) severity: the severity of consequences associated with the outcome; (c) benefits: the belief in the value or efficiency of the change in reducing the susceptibility or severity of the disease [9]; (d) perceived barriers: the barriers to initiate the change (e.g., cost and discomfort); (e) cues to action: the cues for initiating the readiness to begin the change, which include physical/psychological symptoms and increased awareness of the disease (health warning labels/advertisements, the proximity of occurrences of the disease, etc.); and (f) self-efficacy: the motivation/competence to initiate the change [10]. In the context of smoking, an individual will change his/her behaviour if they believe that smoking makes them susceptible to life-threatening conditions, and that effective measures such as smoking cessation programs can help them to quit smoking. Their behaviour changes when they become aware that the change will help reduce the risk and result in better health outcomes.

Studies have shown that smokers understand the risks associated with their behaviour. However, they often underestimate the magnitude of risk [11,12,13]. Harris et al. [12] studied the risk perception among a sample of Bosnian refugees in the US and showed that current smokers tend to disagree with the idea that smokers have a shorter lifespan and are at a higher risk of cancer and other SRDs. The study also reported significant disagreements in the perception of personal risk among smokers, past smokers, and non-smokers, where smokers had a lower perception of the severity of health risk than the rest. Hwang et al. [14] studied a group of smokers and showed that optimistic bias plays a role in this belief. Smokers tend to believe that they are less likely to develop SRDs compared to their peers. The authors reported that participants had a lower relative risk perception despite having the symptoms of SRDs, which was proposed to be due to a lack of awareness of the consequences. Campbell et al. [15] studied the relationship of risk perception to smoking behaviors and showed that a lower risk perception was associated with a higher use of cigarettes and e-cigarettes. Several studies have discussed risk perception in relation to the decision to quit smoking [16,17,18]. A lower risk perception was found to lead to an overestimation of cancer survival rates and negatively affect quitting intentions [16]. Similarly, a lower risk perception among young people was negatively associated with the decision to quit smoking [17,18]. While all these studies focused on risk perception, little attention has been given to other health belief and emotional constructs such as worry, perceived severity, and benefits. These constructs play vital roles in behavioral change related to smoking [19,20].

Health beliefs are mainly shaped through awareness programs [21]. Hsieh et al. [21,22] showed that anti-cigarette campaigns positively impact the knowledge and perception of harm, which was negatively associated with smoking behaviors. Similarly, studies have shown that anti-cigarette information conveyed through various sources such as television, movie theaters, and similar sources can positively shape anti-cigarette attitudes among the viewers [23,24,25].

Individuals with mental illness (MI) smoke twice as much as the general population and have a higher prevalence of nicotine dependence (ND) [26,27]. This is believed to be due to a combination of factors that include genetic vulnerabilities that predispose them to tobacco use, poor coping skills where smoking is used as a method to alleviate the symptoms of MI, and social reinforcement in mental health settings where smoking is considered to be a normal behaviour [28]. MIs are associated with a higher severity of smoking and a lower chance of successful smoking cessation [26,27,28,29,30]. People with MIs have similar motivations to quit smoking as the general population [31]. Nonetheless, the rate of successful quitting is lower due to a higher ND, repeated relapses (due to higher intensity of withdrawal symptoms), a lack of focus on the smoking habits, and therapeutic nihilism [32,33,34]. Most studies to date have compared the risk perception of smokers with that of non-smokers in the general population, and little attention has been given to other health belief constructs and those with MIs. Previous studies have focused on individual constructs in the HBM to predict smoking cessation [12,15,16]. The risk perception construct employed in these studies focused on overall risk, rather than systematically measuring the distinct constructs for absolute and relative risk, thus leading to inconsistencies and, consequently, heterogeneous findings [19]. A systematic measurement of health beliefs is of paramount importance to smoking-related health education and cessation programs. Currently, no such measures have been employed or validated in psychiatric populations to study their smoking-related health beliefs and emotions. Hence, it is crucial to understand the health beliefs of smokers with MIs and the associated factors to identify potential avenues for promoting smoking cessation. This study aimed to understand the (a) health beliefs (perception of risk, benefits, severity, and worry) of smokers and former smokers from a sample of treatment-seeking psychiatric patients, (b) their awareness of sources of anti-smoking information, (c) the factors associated with health beliefs, and (d) the factor structure of the health belief questionnaire.

## 2. Materials and Methods

### 2.1. The Design, Setting, and Sample Size

This cross-sectional study was conducted among treatment-seeking psychiatric patients recruited from the inpatient and outpatient clinics of a tertiary psychiatric care hospital in Singapore. This study derived data from a larger study that established the prevalence and correlations of smoking [27]. A quota sampling method was used with a 1:1 quota assigned based on the diagnosis (depressive disorder, the schizophrenia spectrum, and other psychotic disorders), gender, and age (21–40 and 41–65 years.). A target sample size of 349 was estimated based on the prevalence of current smoking (34.8%) in patients with MIs [26]. With the expectation of 10% missing data, a sample size of 384 was considered sufficient.

### 2.2. Participants

Participants who were between 21 and 65 years of age and had a diagnosis of either depressive disorder, schizophrenia spectrum, or other psychotic disorders were included. Those who did not have the mental capacity to provide written consent were excluded. Participants were recruited through two methods: clinically stable patients were referred by the clinicians to the study team, and self-referral, where participants responded to the recruitment posters displayed in the clinic or the presence of the research staff in the clinic. A trained study team member screened the participants for eligibility and administered the questionnaire face to face. The survey was offered in one of four languages: English, Chinese, Malay, or Tamil, as preferred by the participant. The study procedures were reviewed and approved by the Institution Research Review Committee and National Healthcare Group Domain Specific Review Board, Singapore (Ref: 2018/00772).

### 2.3. Data Collection

#### 2.3.1. Sociodemographic Information

Sociodemographic information such as age, gender, ethnicity, income, occupation, and education were collected from all the participants. Participants who self-reported a lifetime smoking of at least 100 cigarettes and were smoking at the time of the survey were classified as smokers, and those who had smoked 100 cigarettes in their life time and had quit smoking were classified as former smokers [35].Non-smokers were those who had never smoked 100 cigarettes in their life time. 

#### 2.3.2. Smoking-Related Variables

Nicotine dependence was measured using the Fagerstrom Test for ND (FTND; [36]), which is a 6-item scale capturing the participants’ physiological dependence. The binary outcome was used in the analysis where a score of ≥5 was considered as dependence on nicotine [37].

Other smoking-related variables included a family history of smoking, smoking-related health conditions, age of onset, intention, and motivation to quit. A family history of smoking was captured with a single question “Do you have close family members (parents, siblings, or grandparents) who smoke?” The given response options were “yes” or “no” To capture the age of onset of smoking, the participants were asked the question: “How old were you when you first started smoking cigarettes?” Intention to quit [38] was assessed by a single question: “Which of the following describes your action about quitting smoking?” The response options were “I plan to quit in the next 30 days” “I plan to quit in the next 6 months” and “I do not plan to quit in the next 6 months” For the regression model, a binary outcome was used: “I intend to quit within 30 days/6 months” versus “I do not plan to quit in 6 months” Motivation to quit smoking [39] was assessed using a single question for smokers: “How motivated are you to consider quitting smoking?” The response options were “somewhat” “highly motivated and seriously considering” and “not at all motivated” A binary variable was used in the analysis (not at all motivated versus somewhat/highly motivated).

#### 2.3.3. Risk Perception and Other Health Beliefs

The health belief questionnaire consisted of 22 items that were based on the HBM (risk perception, perception of severity, and benefits) and included a construct on emotion (worry) from the self-regulation model (SRM) [19,40]. A modified version of the questionnaire used by the researchers of the study captured responses under four domains: risk perception (risk relative to others: absolute or comparative), worry about lung cancer and other smoking-related diseases (stroke, lung diseases, and heart disease), perceived benefits of adopting the change to reduce the risk, and perceived severity of the consequences of smoking. Smokers and past smokers answered similar questionnaires with two additional questions added to the comparative risk perception module: “Compared with other (current) smokers (replaced the former smokers in the original questionnaire), what do you think is your chance of getting lung cancer in your lifetime?” The total score of the items was calculated by summing up the individual score, where a higher score indicated a higher health belief. Confirmatory factor analysis (CFA) was performed because the questionnaire was not validated within the Singaporean population. The self-efficacy module was removed from the questionnaire because it did not fit well with the other items.

#### 2.3.4. Awareness of the Source of Anti-Smoking Information

A modified version of the ‘media’ module of the Global Adult Tobacco Survey (GAT) [41] was used to measure the awareness of anti-smoking information. Participants were asked, “Have you noticed the health warnings/anti-cigarette information on the following sources?” The sources listed included newspaper/magazines/flyers, television, internet, cinema, and cigarette packets. Answer options were “yes” “no” and “don’t remember” Participants were also asked if “any of the above sources deter them from smoking/effective in discouraging smoking?” An additional question on the effectiveness was asked: “Do you believe that these sources are effective in discouraging people to reduce cigarette consumption?” For regression analysis, a binary variable was used (1 or no sources versus 2 or more sources).

### 2.4. Analysis

Analyses in the present study were conducted with MPlus version 8.2 and Stata version 15. Descriptive statistics are presented as mean ± standard deviation or frequencies for categorical variables. CFA was utilized to evaluate the factor structure of the health belief questionnaire within this sample of current smokers. As the items of the health belief questionnaire were measured on an ordinal scale (i.e., 5-point scale), an unweighted-least-squares with mean- and variance-adjusted (ULSMV option in MPlus) estimator was utilized to model the polychoric correlation matrix (CATEGORICAL option in MPlus). The ULSMV estimator has been shown to be advantageous in small samples of <200 [42]. The following fit indices were used to assess overall model fit and complexity: (i) root mean square error of approximation (RMSEA), (ii) comparative fit index (CFI), (iii) Tucker–Lewis index (TLI), and (iv) standardized root mean square residual (SRMR). Both CFI and TFI values range from 0 to 1, with higher values representing a better fit. CFI values above 0.95 and TLI values above 0.90 are considered to be of excellent fit [43]. Regarding to RMSEA, values below 0.08 indicate moderate fit, while values of 0.05 or less indicate a close fit to the observed data [44]. SRMR values, which indicate an acceptable fit when values are smaller than 0.08 and a good fit when values are smaller than 0.05, were also evaluated [43,44]. Internal consistency was evaluated using Cronbach’s alpha values. Risk perception items were summed to form a total score and their respective factor scores based upon results of the CFA. The differences in risk perception between smokers and former smokers were compared using linear regression models that adjusted for the effects of age and gender. Following this, six linear regressions were conducted to examine the association between risk perception (i.e., total score and individual five factors), sociodemographic, and other smoking-related variables within current smokers only.

## 3. Results

### 3.1. Sociodemographic and Smoking-Related Characteristics

Among the 184 participants (ever smokers) enrolled in the study, 150 (81.5%) were currently smoking and the remaining 34 (18.5%) were former smokers. The majority of the sample were of Chinese ethnicity (63.6%), single (63.0%), and of ages between 41 and 65 years (57.1%). The mean age was 40.6 (±12.0). Approximately half of the participants were unemployed (48.4%) and had a diagnosis of depressive disorder (51.6%). The sociodemographic/smoking-related characteristics of the study population broken down by current and ex-smokers are presented in Table 1. The majority of the sample reported no SRDs (76.6%), and 23.4% reported having at least one of the SRDs. Around 80.9% of the patients reported having a family member who smoked.

### 3.2. Awareness towards Health Warnings/Anti-Cigarette Information

As shown in Table 2, the information on the cigarette packets was identified as the major source of health warnings/anti-cigarette information (95.1%), followed by printed media (62%), TV (60.9%), internet (44.6%), and cinema (37%). Only 31.0% indicated that these sources discouraged them from smoking, while 48% felt that these sources were effective in discouraging smoking in general.

### 3.3. Confirmatory Factor Analysis and Internal Consistency of the Health Belief Questionnaire

CFA was conducted with the ever smokers sample based upon the model put forth by the developers of the health belief questionnaire [40]. However, there were two differences between the model employed by the developers and that of the present study. Firstly, the developers utilized a single item (“How confident are you that you could quit smoking/stay quit for good if you wanted to?”) to assess the construct of “self-efficacy” They thus included this single item in their model as a measured variable rather than a construct. The present study chose to omit this item from the model due to the following reasons: (i) single-item measures tend to be biased, and it is difficult to assess the reliability and validity of the construct being measured [43,44,45]; (ii) the examination of the underlying polychoric correlation matrix revealed that this item had poor correlations with (<0.3) all other items within the matrix; and (iii) a unidimensional exploratory factor analysis with all items showed a factor loading of 0.25, indicating that the item performs poorly in the unidimensional assessment of total risk perception. Secondly, the present study also added two questions: (i) “Compared with other (former) smokers, what do you think is your chance of getting lung cancer in your lifetime?” and (ii) Compared with other (former) smokers, what do you think is your chance of getting a smoking-related disease in your lifetime?” Thirdly, the current study also reworded the original two questions, from comparing to “other smokers” to “current smokers” These four items were specified to load onto the comparative risk factor. Hence, a five-factor solution (absolute risk perception, comparative risk perception, worry, perceived severity, and perceived benefits) was specified. Descriptive information regarding the 24 items can be found in Supplementary Table S1.

CFA results indicated that the five-factor solution had an excellent fit to the observed data (ULSMV χ^2^ = 382.79; df = 220; RMSEA = 0.06; CFI = 0.96; TLI = 0.95; and SRMR = 0.05). No modification indices were specified. The standardized factor loadings of the items to their respective factors were high, ranging from 0.72 to 0.99. The standardized factor loadings of the five-factor solution are displayed in Table 3. The internal consistency (assessed by Cronbach’s alpha values) of the factors was high: absolute risk perception (four-items; α = 0.88), comparative risk perception (eight-items; α = 0.96), worry (four-items; α = 0.92), perceived severity (four-items; α = 0.89), and perceived benefits (three-items; α = 0.86). The internal consistency of all 23-items was also high, with a Cronbach’s alpha value of 0.94.

### 3.4. Differences in Health Beliefs between Current and Former Smokers 

Significant differences in health beliefs were observed between current and former smokers, with current smokers showing significantly higher overall scores (*p* = 0.001). The analysis revealed significantly higher scores in absolute and comparative risk perceptions in current smokers (*p* < 0.001) and a lower perception of benefits (*p* = 0.02). The data are shown in Table 4.

### 3.5. Factors Associated with Risk Perception in Current Smokers

Linear regressions were conducted to examine the associations between health beliefs and sociodemographic and smoking-related information within current-smokers only. Variables that showed a significant association in the univariate analysis were included in the regression.

Age, smoking-related health conditions, ND, intention, and motivation to quit were significantly associated with health beliefs, and all the factors except motivation were also associated with risk perception. Older age groups were significantly associated with lower overall health beliefs (β: −14.1; 95% CI: −22.3, −5.8; *p* = 0.001), absolute risk perception (β: −3.3; 95% CI: −5.1, −1.5; *p* = 0.001), comparative risk perception (β: −6.1; 95% CI: −9.6, −2.5; *p* = 0.001), perceived severity of the consequences (β: −2.4; 95% CI: −4.0, −0.9; *p* = 0.003), and perceived benefits of quitting (β: −1.5; 95% CI: −2.9, −0.1; *p* = 0.04), but not with worry (*p* = 0.50). Surprisingly, awareness and diagnosis were not associated with risk perception. Compared to those with no history of smoking-related diseases, those with at least one condition had significantly higher health beliefs (β: 9.3; 95% CI: 0.7, 18.0; *p* = 0.035), absolute risk perception (β: 1.9; 95% CI: 0.1, 3.8; *p* = 0.04), and worry (β: 2.3; 95% CI: 0.2, 4.4; *p* = 0.03). Nicotine dependence was associated with higher absolute (β: 2.0; 95% CI: 0.2, 3.8; *p* = 0.03) and comparative (β: 4.1; 95% CI: 0.7, 7.6; *p* = 0.02) risk perception.

### 3.6. Risk Perception and Behavioural Intentions

Motivation to quit smoking was associated with higher health beliefs (β: 8.7; 95% CI: 0.6, 16.8; *p* = 0.035), worry (β: 3.1; 95% CI: 1.1, 5.1; *p* = 0.002), perceived severity (β: 1.6; 95% CI: 0.04, 3.1; *p* = 0.045), and perceived benefits of quitting smoking (β: 2.2; 95% CI: 0.9, 3.6; *p* = 0.002). Compared to those planning to quit in the next 30 days/6 months, smokers who had no plans to quit had significantly lower scores on the absolute risk perception (β: −1.9; 95% CI: −3.7, −0.1; *p* = 0.039) factor only. The results of the regression models are indicated in Table 5.

## 4. Discussion

The present study found a good fit and internal consistency of the factor structure of the Health Belief Questionnaire. This indicates that the modified version of the questionnaire is appropriate for the measurement of risk perceptions of smoking amongst smokers with MI. We also found that current smokers with MIs had better overall health beliefs and risk perception (comparative and absolute) than former smokers. Conversely, their perception of the benefits associated with cessation was significantly lower than former smokers. This was consistent with other studies where a higher risk perception was noted among current smokers than in former smokers in a National Lung Trial in the US [20,46]. Smokers understand that they are at a higher risk of mortality than non-smokers and former smokers, and they were knowledgeable about the various risks [47]. The perception of risk improves in former smokers once the behavioral change has been made to stop smoking, as observed in our study (reappraisal hypothesis) [20,38]. A higher risk perception without perceived severity and benefits observed in smokers is a concern because this is associated with a lower likelihood of behaviour change [48]. Risk perception in the absence of perceived benefits can increase fatalistic beliefs (e.g., since SRDs are fatal, quitting is not helpful) that lead to anxiety and lower readiness to quit [49]. This is corroborated by the findings from this study where more than half of the smokers had no immediate plans to quit in the next six months, Hence, it is important to equip smokers with sufficient knowledge of the benefits of smoking cessation and how it impacts their health and wellbeing to encourage a behaviour change.

We also observed that age, history of SRDs, ND, and intention to quit were significantly associated with higher health beliefs. Older age (41–65 years) was associated with lower overall health beliefs, a lower perception of risk (absolute and comparative), benefits, and severity than younger adults (21–40 years). Other studies have reported similar results, where older adults downplayed the risks associated with smoking [50,51,52]. This could be due to the higher optimistic bias observed in older adults [53] as a result of cognitive decline and a sense of immunity based on their past health history (e.g., the belief that smoking did not cause any illness despite smoking for many years) [53,54]. Smoking cessation in older adults carries enormous health benefits, as the risk of mortality and SRDs is significantly reduced upon smoking cessation [55,56]. Additional evidence suggests that older adults have a higher success rate of smoking cessation than younger adults [55,57]. Therefore, this group should be targeted for educational initiatives and smoking cessation to improve their health beliefs.

Smokers with at least one SRD had a better perception of overall risk and significantly higher scores in domains such as absolute risk and worry than those who had no history of SRDs. Borrelli et al. [53] reported that the presence of at least one SRD was associated with risk perception and decision to quit, which was in agreement with our study. Other studies have reported similar results with higher perceived risk and worry among smokers [20,58]. A higher risk perception and worry have been proposed to be important determinants of smoking decision [38] and cancer screening participation [59]. 

ND is strongly associated with a higher perception of risk. This observation is in agreement with the reported studies in smokers [20,53,60,61]. Greillier et al. [60] studied the relationship between ND and risk perception towards cancer and showed that those with ND had a higher risk perception. Similar results were reported by Kowalczyk et al. [61] in a sample of the psychiatric population. Higher risk perception and dependence in the absence of perceived benefits will be a challenge to smoking cessation as groups with these traits experience higher craving, withdrawal, and depressive symptoms during cessation that negatively affect their abstinence [34,48]. Thus, these specific groups will benefit from intense smoking cessation programs with incorporated awareness modules to improve their perception of benefits. 

We observed that the intention to quit smoking was associated with a higher risk perception. Participants who had no plans to quit smoking in the next six months perceived the absolute risk as lower than those who had plans to quit. This was consistent with previous data from the National Lung Trial in the US [20]. Motivation to quit is an important indicator of smoking cessation [62]. In our study, we found that a higher motivation to quit was strongly associated with higher health beliefs, perception of benefits, severity, and worry, which is a strong indicator of a behavioral change. Previous research has corroborated this finding by showing that a lower risk perception is associated with a low motivation to quit, therefore reducing the chances of smoking cessation [53,62]. Furtermore, Layoun et al. [63] showed that higher motivation is associated with a higher readiness to quit. Thus, improving the health benefits through educational/cessation counselling will be beneficial to improve the motivation and readiness to quit.

We also noted that the health warning and anti-cigarette information had no significant effect on risk perception. More than half of the participants reported that this information had no influence on their smoking decision. This was addressed by Lin and Sloan [13], who explained that while this general information may have some effects, it may be ineffective because it is not personalized. The proximity of an event (e.g., seeing a person they knew suffering from SRDs) has a better influence on the participant’s beliefs than general information [13].

The study had several limitations. It was a cross-sectional study that did not give information on the risk perception of former smokers before smoking cessation. Additionally, it was a convenience sample, and care should therefore be taken when generalizing the results to the larger population. The sample size of the former smokers was relatively small and thus a limitation to generalizing the results. Variables such as the age of onset of smoking required the participant to recall the age at which they started smoking, which was subject to recall bias. Though self-efficacy is an important construct in the HBM, we did not assess self-efficacy due to the limited number of items measuring this domain. However, we did examine motivation/intention, which is related to self-efficacy. Given that it is important to the HBM, future studies should improve upon the health belief questionnaire by further developing items for the self-efficacy domain and examining the factor structure of the scale in similar samples. Future research should also examine stigma towards mental illness, which has been shown to be an important factor that can affect smoking behaviors, but was not included in the current questionnaire. 

## 5. Conclusions

Our study found that current and former smokers with MIs have significant differences in their risk perception, as the latter underestimated the risks and benefits. A higher risk perception of the impending dangers of their smoking keeps the person on alert and motivates them to change their behaviour. Age, a history of SRDs, motivation to quit, and intention to quit were associated with health cognition/beliefs that have clinical implications. Clinicians should be aware of these characteristics, and attention should be given to older adults who tend to underestimate the risk and benefits associated with smoking cessation. Understanding the benefits of quitting smoking is important in bringing about behavioral change, which is currently lacking in the study population. Behavioral therapies should include strategies to improve health beliefs and motivation/readiness to quit as they are associated with each other. Targeting the health beliefs of participants with MIs should be prioritized in health communications and cessation counselling. This is achievable through educational initiatives that can be carefully tailored to meet the needs of patients with MI rather than adopting the cessation programs targeted towards the general population.

## Figures and Tables

**Table 1:** Socio-demographic and smoking-related characteristics of the sample. FTND: Fagerstrom Test for nicotine dependence.

Scheme	Current Smokers (n = 150)		Former Smokers (n = 34)	
	n	%	n	%
**Age**				
21–40	61	40.7	18	52.94
41–65	89	59.3	16	47.06
**Gender**				
Male	116	77.3	23	67.7
Female	34	22.7	11	32.4
**Ethnicity**				
Chinese	92	61.3	25	73.5
Malay	32	21.3	5	14.7
Indian	21	14.0	4	11.8
Others	5	3.3	0	0
**Marital Status**				
Single	94	62.7	22	64.7
Married	21	14.0	9	26.5
Divorced/separated/widowed	35	23.3	3	8.8
**Employment Status**				
Employed	60	40.0	21	61.8
Unemployed	78	52.0	11	32.4
Economically inactive	12	8.0	2	5.9
**Clinical Diagnosis**				
Depressive disorder	73	48.7	22	64.7
Schizophrenia spectrum and other psychotic disorder	77	51.3	12	35.3
**Family History of Smoking**				
Yes	119	79.9	29	85.3
No	30	20.1	5	14.7
**Smoking-Related Health Conditions**				
None	118	78.7	23	67.7
At least one	32	21.3	11	32.4
Age of onset				
19 and below	126	84.6	29	85.3
20 and above	23	15.4	5	14.7
**Awareness towards Anti-Cigarette Campaigns**				
1 or less	21	14.0	4	11.8
2 or more	129	86.0	30	88.2
**Nicotine Dependence (FTND)**				
Low or no nicotine dependence (<5)	71	47.7	23	69.7
Has nicotine dependence (≥5)	78	52.4	10	30.3
**Motivation to Quit Smoking**				
Not at all motivated	56	37.3	-	-
Somewhat/Highly motivated	94	62.7	-	-
**Intention to Quit Smoking**				
Plan to quit in 30 days/6 months	65	43.3	-	-
No plans to quit in the next 6months	85	56.7	-	-

**Table 2:** The source of health information and anti-cigarette warning for ever smokers (smokers and former smokers).

Source	Yes	No	Don’t Remember
Newspapers/magazines/flyers	114 (62.0)	54 (29.4)	16 (8.7)
TV	112 (60.9)	61 (33.2)	11 (6.0)
Internet	82 (44.6)	91 (49.5)	11 (6.0)
Cinema	68 (37.0)	99 (53.8)	17 (9.2)
On cigarette packets	175 (95.1)	7 (3.8)	2 (1.1)

**Table 3:** Standardized factor loadings of the five-factor solution amongst ever smokers.

Risk Perception	Estimate
**Absolute Risk Perception**	
How likely do you think it is that you will develop lung cancer in your lifetime?	0.82
How likely do you think it is that you will develop a smoking-related disease in your lifetime?	0.82
I am in danger of developing lung cancer because I smoke.	0.85
I am in danger of developing a smoking-related disease because I smoke.	0.87
**Comparative Risk Perception**	
Compared to others your age and sex, what do you think is your chance of getting lung cancer in your lifetime?	0.90
Compared to others your age and sex, what do you think is your chance of getting a smoking-related disease in your lifetime?	0.89
Compared with other (former) smokers, what do you think is your chance of getting lung cancer in your lifetime?	0.87
Compared with other (current) smokers, what do you think is your chance of getting lung cancer in your lifetime?	0.92
Compared with other (former) smokers, what do you think is your chance of getting a smoking-related disease in your lifetime?	0.86
Compared with other (current) smokers, what do you think is your chance of getting a smoking-related disease in your lifetime?	0.91
I am more in danger of developing lung cancer than the average person.	0.91
I am more in danger of developing a smoking-related disease than the average person.	0.92
**Worry**	
How worried are you about getting lung cancer in your lifetime?	0.96
How worried are you about getting a smoking-related disease in your lifetime?	0.99
How often do you worry about lung cancer?	0.93
How often do you worry about smoking-related diseases (other than lung cancer, such as emphysema, stroke, and heart disease)?	0.81
**Perceived Severity**	
How dangerous do you think lung cancer is?	0.88
How dangerous do you think smoking-related diseases are?	0.88
How serious would the health consequences be if you developed lung cancer?	0.93
How serious would the health consequences be if you developed a smoking-related disease (other than lung cancer, such as emphysema, stroke, and heart disease)?	0.87
**Perceived Benefits**	
In your opinion, how much would quitting smoking reduce your chances of getting lung cancer?	0.97
In your opinion, how much would quitting smoking reduce your chances of getting a smoking-related disease?	0.91
In your opinion, how much would quitting smoking increase your chances of living longer?	0.72
**Factor Correlations**	
Absolute Risk Perception WITH Comparative Risk Perception	0.90
Absolute Risk Perception WITH Worry	0.59
Absolute Risk Perception WITH Perceived Severity	0.44
Absolute Risk Perception WITH Perceived Benefits	0.22
Comparative Risk Perception WITH Worry	0.58
Comparative Risk Perception WITH Perceived Severity	0.48
Comparative Risk Perception WITH Perceived Benefits	0.29
Worry WITH Perceived Severity	0.64
Worry WITH Perceived Benefits	0.47
Perceived Severity WITH Perceived Benefits	0.44

**Table 4:** Comparison of health beliefs among current and former smokers.

Risk Perception Scale Domains	Current Smokers				Former Smokers				B, 95% CI, *p* ^†^
	n	Simple Mean	S.D	Median	n	Simple Mean	S.D	Median	
Total score	149	75.3	21.8	79	33	63.0	12.7	62	B: 13.3, 95% CI: 5.5, 21.1, *p* = 0.001
Absolute risk perception	150	12.9	4.9	13	34	9.1	3.3	9	B: 4.0, 95% CI: 2.3, 5.7, *p* < 0.001
Comparative risk perception	150	25.4	9.3	27.5	33	16.1	6	15	B: 9.8, 95% CI: 6.5, 13.2 *p* < 0.001
Worry	150	10.5	5.3	10	34	8.7	4.9	8	B: 1.8, 95% CI: 0.19, 3.74 *p* = 0.08
Perceived Severity	150	16.8	4.2	18	34	17.6	3.1	18	B: −0.5, 95% CI: −1.93, 1.0, *p* = 0.53
Perceived Benefits	150	9.8	3.7	10	34	11.4	3.7	12	B: −1.7, 95% CI: −3.1, −0.3 *p* = 0.02

^†^ The effects of smoking status (reference group: former smokers) on the risk perception total score and domains were examined in linear regression models that adjusted for the effects of age and gender. B: Unstandardised regression coefficient.

**Table 5:** Factors associated with health beliefs among current smokers.

Predictor Variables	Total Score ^a^		Absolute Risk Perception ^b^		Comparative Risk Perception ^c^		Worry ^d^		Perceived Severity ^e^		Perceived Benefits ^f^	
	Β	95% CI	β	95% CI	β	95% CI	β	95% CI	β	95% CI	β	95% CI
**Age** 21–40 (Ref)												
41–65	−14.1 **	−22.3, −5.8	−3.3 **	−5.1, −1.5	−6.1 *	−9.6, −2.5	−0.7	−2.7, 1.3	−2.4 *	−4.0, −0.9	−1.5 *	−2.9, −0.1
**Gender** Male (Ref)												
Female	2.4	−6.6, 11.3	−0.1	−2.1, 1.8	1.5	−2.3, 5.3	0.5	−1.7, 2.7	1.1	−0.6, 2.8	−0.7	−2.2, 0.8
**Ethnicity**^†^ Chinese (Ref)												
Malay	−5.0	−17.4, 7.4	−0.4	−2.4,1.6	−3.1	−7.0, 0.9	−0.3	−2.5, 2.0	−0.2	−1.9, 1.6	−1.2	−2.8, 0.3
Indian	7.3	−6.6, 21.2	1.4	−0.9, 3.7	1.6	−2.7 6.0	1.6	−0.9, 4.2	2.4	−0.4, 4.3	0.1	−1.6, 1.9
Others	−3.1	−29.7, 23.5	−2.3	−6.6, 2.1	−3.1	−11.5, 5.2	3.6	−1.2, 8.4	0.1	−3.6, 3.9	−1.6	−5.0, 1.7
**Clinical Diagnosis** Depression (Ref)												
Schizophrenia	1.0	−7.2, 9.2	−0.6	−2.5, 1.2	−0.2	−3.7, 3.3	1.0	−1.0, 3.0	−0.2	−1.7, 1.4	1.2	−0.2, 2.6
**Family history of smoking** No (Ref)												
Yes	1.3	−7.6, 10.2	0.1	−1.9, 2.0	1.9	−1.9, 5.7	−0.5	−2.7, 1.7	0.2	−1.5, 1.9	−0.3	−1.8, 1.2
**Awareness towards anti cigarette information** 1 or less sources (Ref)												
2 or more sources	−0.6	−10.6, 9.4	−1.4	−3.6, 0.9	−0.4	−4.7, 3.9	0.3	−2.2, 2.7	−0.04	−2.0, 1.9	1.0	−0.7, 2.7
**Smoking−related health conditions** None (Ref)												
1 or more	9.3 *	0.7, 18.0	1.9 *	0.1, 3.8	2.9	−0.7, 6.5	2.3 *	0.2, 4.4	1.1	−0.5, 2.7	1.4	−0.1, 2.8
**FTND** Low or no nicotine dependence (<5) (Ref)												
Has nicotine dependence (≥5)	7.8	−0.4, 16.0	2.0 *	0.2, 3.8	4.1 *	0.7, 7.6	1.3	−0.8, 3.3	0.2	−1.3, 1.8	−0.1	−1.5, 1.3
**Age of onset** 19 and below (Ref)												
20 and above	2.8	−7.0, 12.6	0.5	−1.7, 2.7	−0.9	−5.0, 3.3	0.5	−1.9, 2.9	0.9	−1.0, 2.8	1.6	−0.04,3.3
**Motivation to quit smoking** Not at all motivated (Ref)												
Somewhat/Highly motivated	8.7 *	0.6, 16.8	0.6	−1.2, 2.4	1.2	−2.3, 4.6	3.1 **	1.1, 5.1	1.6 *	0.04, 3.1	2.2 **	0.9, 3.6
**Intention to quit smoking** Plan to quit in 30 days/6 months (Ref)												
No plans to quit in next 6months	−5.7	−13.8, 2.4	−1.9 *	−3.7, −0.1	−3.1	−6.5, 0.4	−0.3	−2.3, 1.6	−0.5	−2.1, 1.0	0.03	−1.4, 1.4

^a^ Total risk perception, n = 149, mean = 75.32 ± 21.8, range 23–115; ^b^ absolute risk perception, n = 150, mean = 12.9 ± 4.9, range 4–20; ^c^ comparative risk perception, n = 150, mean = 25.4 ± 9.3, range 8–40; ^d^ worry, n = 149, mean = 10.5 ± 5.3, range 4–20; ^e^ perceived severity, n = 150, mean = 16.8 ± 4.2, range 4–20; ^f^ perceived benefits, n = 150, mean = 9.8 ± 3.7, range 3–15. ^†^ The estimates for ethnicity were adjusted with Bonferroni correction for multiple comparisons benefits, * *p* < 0.05, and ** *p* < 0.01.

## Data Availability

The data presented in this study are available on request from the corresponding author. The data are not publicly available due to institutional policies.

## Supplementary Materials

The following are available online at https://www.mdpi.com/1660-4601/18/4/1571/s1, Table S1: Descriptive information for the risk perception scale.
