# Supplementary material for: Smoking-Related Health Beliefs in a Sample of Psychiatric Patients: Factors Associated with the Health Beliefs and Validation of the Health Belief Questionnaire
Source: Int J Environ Res Public Health. 2021 Feb 7;18(4):1571. doi: 10.3390/ijerph18041571 (PMC7916053; doi:10.3390/ijerph18041571)
Supplement: Supplementary file 1 [file ijerph-18-01571-s001.pdf]

**Supplementary section**

| | | Smokers | | | | | Ex-Smokers | | | | |
|---|---|---|---|---|---|---|---|---|---|---|---|
| No. | Item description | **Very unlikely** | | | | **Very likely** | **Very unlikely** | | | | **Very likely** |
| 1 | How likely do you think it is that you will develop lung cancer in your lifetime? | 38<br>25.3% | 18<br>12.0% | 39<br>26.0% | 27<br>18.0% | 28<br>18.7% | 11<br>32.4% | 14<br>41.2% | 7<br>20.6% | 2<br>5.9% | 0<br>0.0% |
| 2 | How likely do you think it is that you will develop a smoking-related disease in your lifetime? | 26<br>17.3% | 20<br>13.3% | 32<br>21.3% | 41<br>27.3% | 31<br>20.7% | 10<br>29.4% | 13<br>38.2% | 9<br>26.5% | 2<br>5.9% | 0<br>0.0% |
| | | **Strongly disagree** | | | | **Strongly agree** | **Strongly disagree** | | | | **Strongl y agree** |
| 3 | I am in danger of developing lung cancer because I smoke | 32<br>21.3% | 16<br>10.7% | 27<br>18.0% | 31<br>20.7% | 44<br>29.3% | 9<br>26.5% | 8<br>23.5% | 11<br>32.4% | 4<br>11.8% | 2<br>5.9% |
| 4 | I am in danger of developing a smoking-related disease because I smoke. | 22<br>14.7% | 16<br>10.7% | 29<br>19.3% | 36<br>24.0% | 47<br>31.3% | 7<br>20.6% | 11<br>32.4% | 8<br>23.5% | 6<br>17.7% | 2<br>5.9% |
| | | **Much lower** | | | | **Much higher** | **Much lower** | | | | **Much higher** |
| 5 | Compared to others your age and sex, what do you think is your chance of getting lung cancer in your lifetime? | 27<br>18.0% | 15<br>10.0% | 42<br>28.0% | 36<br>24.0% | 30<br>20.0% | 10<br>29.4% | 9<br>26.5% | 12<br>35.3% | 2<br>5.9% | 1<br>2.9% |
| 6 | Compared to others your age and sex, what do you think is your chance of | 22<br>14.7% | 19<br>12.7% | 43<br>28.7% | 39<br>26.0% | 27<br>18.0% | 9<br>26.5% | 10<br>29.4% | 13<br>38.2% | 1<br>2.9% | 1<br>2.9% |

| | | | | | | | | | | | |
|---|---|---|---|---|---|---|---|---|---|---|---|
| | getting a smoking-related disease in your lifetime? | | | | | | | | | | |
| 7 | Compared with other (former) smokers, what do you think is your chance of getting lung cancer in your lifetime? | 17<br>11.3% | 24<br>16.0% | 47<br>31.3% | 28<br>18.7% | 34<br>22.7% | 10<br>29.4% | 14<br>41.2% | 8<br>23.5% | 2<br>5.9% | 0<br>0.0% |
| 8 | Compared with other (current) smokers, what do you think is your chance of getting lung cancer in your lifetime? | 26<br>17.3% | 18<br>12.0% | 61<br>40.7% | 24<br>16.0% | 21<br>14.0% | 19<br>57.6% | 9<br>27.3% | 3<br>9.1% | 2<br>6.1% | 0<br>0.0% |
| 9 | Compared with other (former) smokers, what do you think is your chance of getting a smoking-related disease in your lifetime? | 20<br>13.3% | 22<br>14.7% | 42<br>28.0% | 41<br>27.3% | 25<br>16.7% | 12<br>35.3% | 13<br>38.2% | 7<br>20.6% | 2<br>5.9% | 0<br>0.0% |
| 10 | Compared with other (current) smokers, what do you think is your chance of getting a smoking-related disease in your lifetime? | 23<br>15.3% | 22<br>14.7% | 61<br>40.7% | 21<br>14.0% | 23<br>15.3% | 19<br>57.6% | 8<br>24.2% | 5<br>15.2% | 1<br>3.0% | 0<br>0.0% |
| 11 | I am more in danger of developing lung cancer than the average person. | 29<br>19.3% | 18<br>12.0% | 15<br>10.0% | 52<br>34.7% | 36<br>24.0% | 14<br>41.2% | 6<br>17.7% | 10<br>29.4% | 4<br>11.8% | 0<br>0.0% |
| 12 | I am more in danger of developing a smoking-related disease than the average person. | 24<br>16.0% | 23<br>15.3% | 21<br>14.0% | 44<br>29.3% | 38<br>25.3% | 11<br>32.4% | 9<br>26.5% | 11<br>32.4% | 3<br>8.8% | 0<br>0.0% |
| | | **Not at all** | | | | **Extremely** | **Not at all** | | | | **Extremely** |
| 13 | | 42 | 23 | 30 | 23 | 31 | 15 | 8 | 3 | 5 | 3 |

| | | | | | | | | | | | |
|---|---|---|---|---|---|---|---|---|---|---|---|
| | How worried are you about getting lung cancer in your lifetime? | 28.2% | 15.4% | 20.1% | 15.4% | 20.8% | 44.1% | 23.5% | 8.8% | 14.7% | 8.8% |
| 14 | How worried are you about getting a smoking-related disease in your lifetime? | 44<br>29.3% | 26<br>17.3% | 28<br>18.7% | 24<br>16.0% | 28<br>18.7% | 12<br>35.3% | 11<br>32.4% | 3<br>8.8% | 5<br>14.7% | 3<br>8.8% |
| 15 | How often do you worry about lung cancer? | 60<br>40.0% | 26<br>17.3% | 29<br>19.3% | 14<br>9.3% | 21<br>14.0% | 18<br>52.9% | 7<br>20.6% | 4<br>11.8% | 1<br>2.9% | 4<br>11.8% |
| 16 | How often do you worry about smoking-related diseases (other than lung cancer, such as emphysema, stroke, and heart disease)? | 51<br>34.0% | 30<br>20.0% | 28<br>18.7% | 21<br>14.0% | 20<br>13.3% | 13<br>38.2% | 11<br>32.4% | 3<br>8.8% | 3<br>8.8% | 4<br>11.8% |
| 17 | How dangerous do you think lung cancer is? | 10<br>6.7% | 6<br>4.0% | 14<br>9.3% | 15<br>10.0% | 105<br>70.0% | 0<br>0.0% | 1<br>2.9% | 2<br>5.9% | 5<br>14.7% | 26<br>76.5% |
| 18 | How dangerous do you think smoking-related diseases are? | 11<br>7.3% | 9<br>6.0% | 24<br>16.0% | 36<br>24.0% | 70<br>46.7% | 1<br>2.9% | 0<br>0.0% | 6<br>17.7% | 9<br>26.5% | 18<br>52.9% |
| 19 | How serious would the health consequences be if you developed lung cancer? | 7<br>4.7% | 8<br>5.3% | 13<br>8.7% | 14<br>9.3% | 108<br>72.0% | 1<br>2.9% | 2<br>5.9% | 0<br>0.0% | 9<br>26.5% | 22<br>64.7% |
| 20 | How serious would the health consequences be if you developed a smoking-related disease (other than lung cancer, such as emphysema, stroke, and heart disease)? | 9<br>6.0% | 7<br>4.7% | 24<br>16.0% | 26<br>17.3% | 84<br>56.0% | 2<br>5.9% | 0<br>0.0% | 2<br>5.9% | 14<br>41.2% | 16<br>47.1% |
| 21 | In your opinion, how much would quitting smoking reduce your chances of getting lung cancer? | 26<br>17.3% | 17<br>11.3% | 40<br>26.7% | 24<br>16.0% | 43<br>28.7% | 3<br>8.8% | 2<br>5.9% | 8<br>23.5% | 7<br>20.6% | 14<br>41.2% |

| | | | | | | | | | | | |
|---|---|---|---|---|---|---|---|---|---|---|---|
| 22 | In your opinion, how much would quitting smoking reduce your chances of getting a smoking-related disease? | 24<br>16.0% | 15<br>10.0% | 39<br>26.0% | 34<br>22.7% | 38<br>25.3% | 3<br>8.8% | 2<br>5.9% | 9<br>26.5% | 5<br>14.7% | 15<br>44.1% |
| 23 | In your opinion, how much would quitting smoking increase your chances of living longer? | 30<br>20.0% | 19<br>12.7% | 37<br>24.7% | 22<br>14.7% | 42<br>28.0% | 3<br>8.8% | 2<br>5.9% | 8<br>23.5% | 7<br>20.6% | 14<br>41.2% |
| 24* | How confident are you that you could quit smoking/stay quit for good if you wanted to? | 33<br>22.0% | 33<br>22.0% | 25<br>16.7% | 29<br>19.3% | 30<br>20.0% | 2<br>5.9% | 0<br>0.0% | 2<br>5.9% | 7<br>20.6% | 23<br>67.7% |

*This item was not included in total or factor scores
